# Postponed or immediate drainage of infected necrotizing pancreatitis (POINTER trial): study protocol for a randomized controlled trial

**DOI:** 10.1186/s13063-019-3315-6

**Published:** 2019-04-25

**Authors:** Janneke van Grinsven, Sven M. van Dijk, Marcel G. Dijkgraaf, Marja A. Boermeester, Thomas L. Bollen, Marco J. Bruno, Sandra van Brunschot, Cornelis H. Dejong, Casper H. van Eijck, Krijn P. van Lienden, Djamila Boerma, Peter van Duijvendijk, Muhammed Hadithi, Jan Willem Haveman, René W. van der Hulst, Jeroen M. Jansen, Daan J. Lips, Eric R. Manusama, I. Quintus Molenaar, Donald L. van der Peet, Alexander C. Poen, Rutger Quispel, Alexander F. Schaapherder, Erik J. Schoon, Matthijs P. Schwartz, Tom C. Seerden, B. W. Marcel Spanier, Jan Willem Straathof, Niels G. Venneman, Wim van de Vrie, Ben J. Witteman, Harry van Goor, Paul Fockens, Hjalmar C. van Santvoort, Marc G. Besselink, for the Dutch Pancreatitis Study Group

**Affiliations:** 1 Department of Surgery, Amsterdam UMC, University of Amsterdam, G4.196, PO Box 26000, 1105 AZ Amsterdam, Netherlands; 2 Department of Surgery, St. Antonius Hospital Nieuwegein, Nieuwegein, Netherlands; 3 Clinical Research Unit, Amsterdam UMC, University of Amsterdam, Amsterdam, Netherlands; 4 Department of Radiology, St. Antonius Hospital Nieuwegein, Nieuwegein, Netherlands; 5 Department of Gastroenterology and Hepatology, Erasmus MC University Medical Center Rotterdam, Rottedam, Netherlands; 6 Department of Gastroenterology and Hepatology, Amsterdam UMC, University of Amsterdam, Amsterdam, Netherlands; 7 Department of Surgery, University Medical Center Utrecht, Cancer Center, Utrecht, Netherlands; 8 Department of Surgery, Maastricht University Medical Center+, Maastricht, Netherlands; 9 NUTRIM School for Nutrition and Translational Research in Metabolism, Maastricht, Netherlands; 10 Department of Surgery, Erasmus MC University Medical Center Rotterdam, Rotterdam, Netherlands; 11 Department of Radiology, Amsterdam UMC, University of Amsterdam, Amsterdam, Netherlands; 12 Department of Surgery, Gelre Hospital Apeldoorn, Apeldoorn, Netherlands; 13 Department of Gastroenterology and Hepatology, Maasstad Hospital Rotterdam, Rotterdam, Netherlands; 14 Department of Surgery, University Medical Center Groningen, University of Groningen, Groningen, Netherlands; 15 Department of Gastroenterology and Hepatology, Spaarne Gasthuis Haarlem, Haarlem, Netherlands; 16 Department of Gastroenterology and Hepatology, OLVG Amsterdam, Amsterdam, Netherlands; 17 Department of Surgery, Jeroen Bosch Hospital, ’s-Hertogenbosch, Netherlands; 18 Department of Surgery, Medical Center Leeuwarden, Leeuwarden, Netherlands; 19 Department of Surgery, Amsterdam UMC, Vrije Universiteit Amsterdam, Amsterdam, Netherlands; 20 Department of Gastroenterology and Hepatology, Isala Clinics Zwolle, Zwolle, Netherlands; 21 Department of Gastroenterology and Hepatology, Reinier de Graaf Gasthuis Delft, Delft, Netherlands; 22 Department of Surgery, Leids University Medical Center Leiden, Leiden, Netherlands; 23 Department of Gastroenterology and Hepatology, Catharina Hospital Eindhoven, Eindhoven, Netherlands; 24 Department of Gastroenterology and Hepatology, Meander Medical Center Amersfoort, Amersfoort, Netherlands; 25 Department of Gastroenterology and Hepatology, Amphia Hospital Breda, Breda, Netherlands; 26 Department of Gastroenterology and Hepatology, Rijnstate Hospital Arnhem, Arnhem, Netherlands; 27 Department of Gastroenterology and Hepatology, Maxima Medical Center Veldhoven, Veldhoven, Netherlands; 28 Department of Gastroenterology and Hepatology, Medisch Spectrum Twente Enschede, Enschede, Netherlands; 29 Department of Gastroenterology and Hepatology, Albert Schweitzer Hospital Dordrecht, Dordrecht, Netherlands; 30 Department of Gastroenterology and Hepatology, Hospital Gelderse Vallei Ede, Ede, Netherlands; 31 Department of Surgery, Radboud University Medical Center Nijmegen, Nijmegen, Netherlands

**Keywords:** Necrotizing pancreatitis, Infection, Step-up approach, Timing, Drainage, Complication, Randomized controlled trial

## Abstract

**Background:**

Infected necrosis complicates 10% of all acute pancreatitis episodes and is associated with 15–20% mortality. The current standard treatment for infected necrotizing pancreatitis is the step-up approach (catheter drainage, followed, if necessary, by minimally invasive necrosectomy). Catheter drainage is preferably postponed until the stage of walled-off necrosis, which usually takes 4 weeks. This delay stems from the time when open necrosectomy was the standard. It is unclear whether such delay is needed for catheter drainage or whether earlier intervention could actually be beneficial in the current step-up approach. The POINTER trial investigates if immediate catheter drainage in patients with infected necrotizing pancreatitis is superior to the current practice of postponed intervention.

**Methods:**

POINTER is a randomized controlled multicenter superiority trial. All patients with necrotizing pancreatitis are screened for eligibility. In total, 104 adult patients with (suspected) infected necrotizing pancreatitis will be randomized to immediate (within 24 h) catheter drainage or current standard care involving postponed catheter drainage. Necrosectomy, if necessary, is preferably postponed until the stage of walled-off necrosis, in both treatment arms. The primary outcome is the Comprehensive Complication Index (CCI), which covers all complications between randomization and 6-month follow up. Secondary outcomes include mortality, complications, number of (repeat) interventions, hospital and intensive care unit (ICU) lengths of stay, quality-adjusted life years (QALYs) and direct and indirect costs. Standard follow-up is at 3 and 6 months after randomization.

**Discussion:**

The POINTER trial investigates if immediate catheter drainage in infected necrotizing pancreatitis reduces the composite endpoint of complications, as compared with the current standard treatment strategy involving delay of intervention until the stage of walled-off necrosis.

**Trial registration:**

ISRCTN, 33682933. Registered on 6 August 2015. Retrospectively registered.

**Electronic supplementary material:**

The online version of this article (10.1186/s13063-019-3315-6) contains supplementary material, which is available to authorized users.

## Background

Acute pancreatitis is one of the most common gastrointestinal conditions requiring acute hospital admission [1]. Around 20–30% of these patients develop necrotizing pancreatitis [2]. Infected necrotizing pancreatitis occurs in a third of these patients and is associated with 15–20% mortality [3, 4], despite radiological, endoscopic or surgical interventions [3–6]. The current, 2013 international treatment guidelines [7, 8] recommend a step-up approach, based on the results of the Dutch PANTER trial [3]. The first step of this step-up approach is catheter drainage, preferably once the (extra) pancreatic collection has organized and has become fully encapsulated (walled-off necrosis). This process is usually complete 4 weeks after the onset of disease. During this time, intravenous antibiotic treatment is used which may obviate the need for any intervention in a small subset of patients [4]. If catheter drainage does not resolve the clinical signs of infection and sepsis, surgical [3] or endoscopic [9] necrosectomy is performed as the next step.

Postponing all interventions for infected necrosis until the stage of walled-off necrosis has been standard practice for many years. The rationale for this delay lies in the prevention of the “extra hit” (i.e. a pro-inflammatory reaction) of open surgery in these already critically ill patients, and in the relationship between early open necrosectomy and mortality [10]. In line with this practice, catheter drainage in the current step-up approach has also been postponed until the stage of walled-off necrosis. Meanwhile, intravenous antibiotics are administered to reduce systemic illness from the infected necrosis, which may lead to increased incidence of Candida infections and antibiotic resistance [10]. Notably, several observational studies have suggested that encapsulation is not mandatory for safe and successful catheter drainage [3, 11–15]. In other conditions, such as pancreatic fistula after pancreatic resection, early (percutaneous) catheter drainage has also proven to be safe and successful [16]. Furthermore, an international survey among expert pancreatologists demonstrated “equipoise” between immediate and postponed catheter drainage of infected necrotizing pancreatitis [17]. The aim of immediate catheter drainage is to prevent further clinical deterioration.

## Methods

**Fig. 1:**
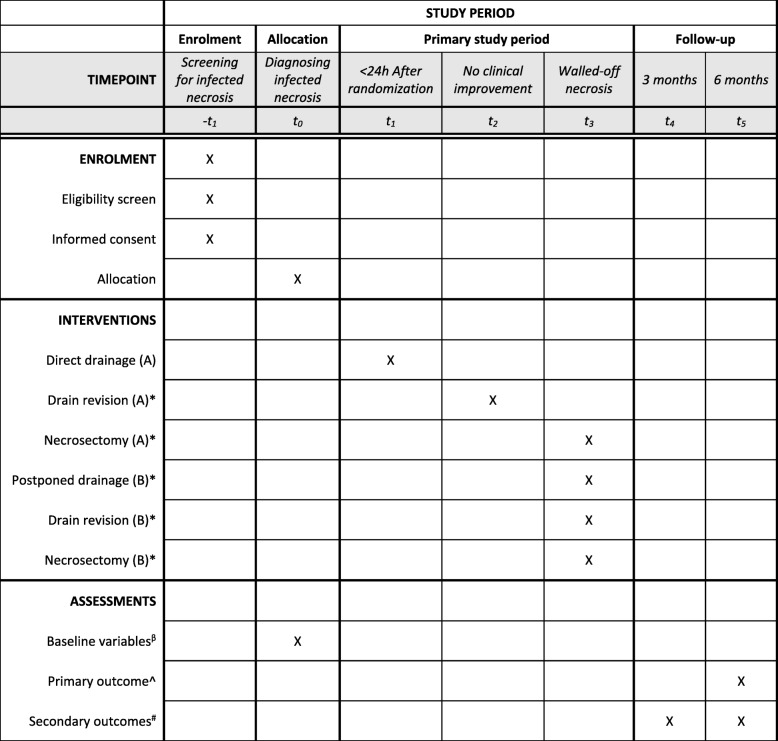
SPIRIT schedule of enrollment, interventions, and assessments. *In the case of no clinical improvement. ^β^Baseline variables: age, sex, center, body mass index, etiology of pancreatitis, American Society of Anesthesiologist’s classification, co-morbidity, disease severity, suspected or proven infected necrosis, time from admission to randomization, time from admission to tertiary referral. ^Primary outcome: Comprehensive Complications Index. ^#^Secondary outcomes: mortality, complications, number of (repeat) interventions, hospital and intensive care unit lengths of stay, quality-adjusted life years and direct and indirect costs

The trial protocol is written in accordance with the Standard protocol items: recommendation for interventional trials (SPIRIT) guidelines (see Fig. 1 and the Additional file 1: SPIRIT checklist) [18].

### Study aim

The POINTER trial aims to determine whether immediate catheter drainage in patients with (suspected) infected necrotizing pancreatitis is superior to the current standard of postponed catheter drainage with regard to clinical outcome and cost-effectiveness. The hypothesis is that pro-active diagnosis of infected necrosis and immediate catheter drainage prevents further clinical deterioration in these patients, reducing complications and possibly death, and reduces length of hospital stay and costs, as compared to postponing catheter drainage using antibiotics, preferably until the stage of walled-off necrosis.

### Study design and setting

POINTER is a randomized controlled multicenter superiority trial, including hospitalized adult patients with proven or suspected infected necrotizing pancreatitis. In total, 25 centers are participating in the trial, including all 8 Dutch university medical centers. Endpoints are assessed by an adjudication committee, blinded to the assigned treatment arm, based on clinical case descriptions and endpoint definitions. The Data and Safety Monitoring Committee (DSMC) monitors patient safety.

#### Inclusion criteria (see Additional file 2: Table S1 and Additional file 3: Table S2)

The inclusion criteria are:

- Proven infected necrotizing pancreatitis (0–35 days after the onset of disease) or clinical suspected infected necrosis (15–35 days after the onset of disease)
- Catheter drainage of the necrotic collection is technically feasible, as deemed by the expert panel and/or treating physician (i.e. enough encapsulation and liquefaction)
- Age ≥ 18 years

#### Exclusion criteria

The exclusion criteria are:

- Onset of acute pancreatitis > 35 days ago
- Indication for emergency laparotomy because of an abdominal catastrophe (e.g. bleeding, bowel perforation or abdominal compartment syndrome)
- Previous retroperitoneal intervention for necrotizing pancreatitis (ascites drainage is permitted and (emergency) laparotomy without opening the bursa is permitted)
- Documented chronic pancreatitis (according to the M-ANNHEIM criteria [36], since this is a different disease entity)

### Treatment groups

All patients with signs of infected necrotizing pancreatitis are pro-actively assessed for the presence of infected necrosis, either by imaging (gas configurations), fine-needle aspiration (FNA), or clinical signs of infection, which may include persistent organ failure. See Additional file 4: Figure S1 for the inclusion flowchart. FNA is only used on indication and not as a screening tool. Patients who fulfill the eligibility criteria are randomly assigned to group A or B. Since diagnosing infected necrosis and making decisions on whether to perform invasive intervention in these patients are challenging, the Dutch pancreatitis expert panel [19] is 24 hours per day, 7 days per week, to assess indications for intervention and eligibility for randomization as was done in the PANTER [3] and TENSION [9] trials.

### Intervention group (A)

Group A will receive immediate catheter drainage within 24 h after randomization, while starting (or continuing) antibiotic treatment. In the case of no clinical improvement within 72 h thereafter, the possibility of additional drainage is evaluated, including drain revision or drain upsizing. In the case of no clinical improvement thereafter, and no possibilities for additional catheter drainage, minimally invasive necrosectomy is performed once the (extra) pancreatic collection has developed into walled-off necrosis. No clinical improvement is defined as new organ failure or 2/3 parameters that do not decrease (temperature, C-reactive protein (CRP) and leukocyte count). See flowchart in Additional file 4: Figure S1.

### Control group (B)

Group B will have postponed catheter drainage and receive antibiotics, preferably until the (extra) pancreatic necrotic collection has reached the stage of walled-off necrosis. In case of no clinical improvement within 72 h after drainage, the possibility of additional drainage is evaluated, including drain revision or drain upsizing. In case of no clinical improvement thereafter and no possibilities for additional catheter drainage, minimally invasive necrosectomy is performed. No clinical improvement is defined as new organ failure or 2/3 parameters that do not decrease (temperature, CRP and leukocyte count). See flowchart in Additional file 4: Figure S1.

### Diagnosing infected necrosis

All patients with signs of infected necrotizing pancreatitis are screened for eligibility (Additional file 4: Figure S1). We differentiate between diagnosis of infected necrosis within the first 14 days after onset of disease and diagnosis thereafter. In the first 14 days a proven infection is mandatory, as in this early course of the disease it is impossible to distinguish systemic inflammatory response syndrome (SIRS) from sepsis. This proof requires either a positive gram stain or culture from FNA or gas configurations in the (peri) pancreatic collection with necrosis, on imaging (contrast enhanced computed tomography (CECT) or magnetic resonance imaging (MRI)). In the case of unclear signs of infection, percutaneous FNA is performed in patients with necrotizing pancreatitis within 14 days after onset of disease who have had clinical signs of infection (i.e. new (multiple) organ failure or 2/3 parameters raised: temperature, CRP or leukocyte count) for 2 days consecutively . After the first 14 days, clinical signs of infection suffice for diagnosing infected necrosis (having no other focus for infection, e.g. pneumonia), based on our experiences in previous trials [3, 9]. Obviously, in the case of clinical doubt, FNA is still allowed after the first 14 days. The presence of gas in the (extra) pancreatic necrosis on CECT is considered proven infected necrosis in all patients, regardless of the disease stage (i.e. before or after 14 days).

### Supportive treatment

Current standard management of acute (necrotizing) pancreatitis is extensively described in the International Association of Pancreatology (IAP)/American Pancreatic Association (APA) guidelines of 2013 [7]. These international guidelines have been adopted by the Dutch Pancreatitis Study Group (DPSG) and the national professional organizations involved. In accordance with these guidelines, patients receive fluid resuscitation, pain management and enteral feeding (or oral, if enteral feeding is not tolerated). Antibiotic prophylaxis is not given. The source of infection will be investigated in patients with clinical signs of infection and deterioration, by means of analysis of blood, urine, sputum, and ascites and by diagnostic imaging (e.g. chest x-ray and abdominal CECT). Once the focus of infection is identified, targeted antibiotics are given or, in when there infected necrosis is suspected and there is persistent deterioration, broad spectrum antibiotics with optimal tissue penetration are started empirically. The latter usually consists of meropenem or imipenem, based on the local antibiotics protocol.

### Step-up approach

Both a percutaneous surgical and an endoscopic step-up approach are permitted in the POINTER trial, depending on the location of the necrotic collection(s), the extent of encapsulation and the preference of the treating physician. Transluminal (transgastric or transduodenal), endoscopic or percutaneous catheter drainage via the retroperitoneal route may be performed. In the case of no clinical improvement within the first 72 h after initial catheter drainage, an additional or upsizing drainage procedure is performed (Additional file 4: Figure S1). Necrosectomy is performed when there is no clinical improvement after this second drainage procedure and no further possibilities for optimized or additional drainage. After percutaneous catheter drainage, additional necrosectomy should be performed surgically (e.g. videoscopic assisted retroperitoneal debridement (VARD)) and after endoscopic transluminal drainage, endoscopic transluminal necrosectomy is performed. Additional catheter drainage or necrosectomy may be performed using the “other” approach only when (additional) necrotic collections are not technically approachable using the standard second step. Both step-up approaches are performed to conform with the PANTER and TENSION trial protocols [3, 9]; see also Additional file 5: Figure S2 and Additional file 6: Figure S3. Randomized studies can be influenced by underestimation of learning curves, therefore in the POINTER trial the participating centers must have documented expertise, defined as having performed at least 10 independent VARD procedures, 10 independent endoscopic transluminal drainage procedures and 10 independent endoscopic transluminal necrosectomies. This number has been chosen to achieve a balance between volume and feasibility, and from experience with other trials of our study group including necrotizing pancreatitis patients [3, 9]. In the case of there being insufficient local experience available (e.g. during weekends), the patient is transferred to a tertiary referral center with sufficient experience.

### Primary endpoint

The primary endpoint is the Comprehensive Complications Index (CCI) [20, 21], including all complications other than pre-existent complications (e.g. treatment for infected (extra) pancreatic necrosis) occurring after randomization until 6 months after randomization, and graded according to the Clavien-Dindo classification. These complications are assessed by an adjudication committee, blinded for assigned treatment arm, based on clinical case descriptions using definitions.

### Secondary endpoints

Secondary endpoints are mortality, new-onset (multiple) organ failure, bleeding requiring intervention, perforation of a visceral organ requiring intervention, enterocutaneous and pancreatic fistula, incisional hernia (including burst abdomen), wound infections, endocrine and exocrine pancreas insufficiency, number of patients with severe complications (Clavien-Dindo III or higher), number of patients per Clavien-Dindo classification, number of surgical, endoscopic and radiological (repeat) interventions, length of hospital stay, length of ICU admission, quality-adjusted life years (QALYs) and total direct and indirect costs (see Additional file 7 for relevant definitions). Also, for mutual comparison, the primary endpoint of the previous PANTER trial will be a secondary endpoint of the POINTER trial (i.e. a composite of major complications: new-onset multiple organ failure, enterocutaneous fistula or perforation of a visceral organ requiring intervention, intra-abdominal bleeding requiring intervention or death during admission or during the 3 months after discharge) [3].

### Sample size

The sample size was calculated based on the primary endpoint, the CCI. A mean CCI score of 40 (with standard deviation of 27) for postponed catheter drainage is based on the number of complications identified in the step-up arm of the PANTER trial [3] and TENSION trial [9]. Analysis by Student’s *t* test will have 80% power to detect a clinically relevant reduction of 15 to a CCI score of 25 [21] at a significance level of 0.05; for a sample size that equals 2 × 51, this will result in 102 evaluable patients. Assuming a dropout rate of about 2%, then 104 patients need to be included. A drop-out rate of 2% is relatively low, but was chosen from experience with other trials in our study group including patients with necrotizing pancreatitis, in which no there was no loss to follow-up or drop-outs [3, 9].

### Randomization, blinding and treatment allocation

Patients are randomized using a centrally operated computer (ALEA system) with variable block randomization for allocation concealment between group A (immediate catheter drainage) and group B (postponed catheter drainage). Stratification at randomization is applied for the following factors: presence of organ failure (yes versus no), disease duration (day 0–20 versus day 21–35) and center (expected high number of included patients versus other centers). For randomization of a patient, physicians can contact the study coordinator via telephone (www.pancreatitis.nl) 24 h per day, 7 days per week in order to check the eligibility criteria and to verify whether informed consent has been obtained. Blinding of patients and physicians to treatment strategy is not feasible, since both treatments are highly different timing-wise. Patients are coded by a numeric randomization code (anonymized).

### Follow up

The follow-up duration is 6 months from randomization. Outpatient follow-up visits take place at the discretion of the responsible physician, but in any case, follow up at 3 and 6 months after randomization can be considered as standard care. All patients undergo imaging (preferably CECT) at 3 and 6 months post randomization. Furthermore, exocrine and endocrine pancreatic function is measured at these points in time, with blood glucose measurements and fecal elastase tests, respectively. No blood or fecal samples are stored at the DPSG data center. The treating physician is responsible for the application, interpretation and treatment when needed. Also, every patient receives a combined questionnaire at home (Short Form-36 (SF-36) [22], Euroqol 5 dimensions (EQ-5D) [23], iMedical Consumption Questionnaire (iMCQ) [24] and iProductivity Cost Questionnaire (iPCQ) [25]) at 3 and 6 months. Data from patient records are collected until 6 months post randomization.

### Baseline values

Baseline criteria (all < 24 h prior to randomization) are age, sex, center, body mass index, etiology of pancreatitis, American Society of Anesthesiologist’s (ASA) classification, co-morbidity, disease severity (SIRS, ICU admission, single or multiple organ failure, Acute Physiology and Chronic Health Evaluation (APACHE) II score, Multiple Organ Dysfunction Score (MODS) [26], Sequential Organ Failure Assessment (SOFA) score [27], CRP), CT severity index (CTSI), suspected or proven infected necrosis, time from admission to randomization (days) and time from admission to tertiary referral (days).

### Statistical analysis

All randomized patients are evaluated for primary and secondary endpoints at 6 months after randomization. Using primary source data, an adjudication committee will assess the occurrence of the primary and secondary outcomes blinded to treatment allocation, after the last patient has completed the predefined follow up 6 months after randomization.

The primary analysis is based on intention-to-treat principles. For exploratory reasons per-protocol analysis will also performed. A tabular listing of all patients excluded from the intention-to-treat populations will be provided together with the reasons for exclusion. For the intention-to-treat population the protocol deviations in each randomization arm are listed. Predefined subgroup analyses will be performed in patients with and without (multiple) organ failure and disease duration (cutoff 20 days) at the time of randomization and per center (high expected number of included patients and other centers).

All analyses will be performed in SPSS for Windows or SAS System for Windows. All data handling and analyses will be saved in a syntax/program file. Results will be presented with all centers combined. A two-tailed *p* value <0.05 is considered statistically significant. No corrections for multiple tests will be applied.

The primary outcome is a sum of all complications that are weighted for their severity (multiplication of the median reference values from patients and physicians), the CCI [20, 21]. Comparison of the primary endpoint will be expressed in terms of the absolute difference in mean CCI score and standard deviation (SD). Subsequent analyses will be directed at the secondary endpoints. Data will be presented as mean ± SD and in the case of skewed distributions as median and range. Values will be compared using Student’s *t* test, Wilcoxon’s rank sum test, the chi square (χ2) test or Fischer;s exact test, as appropriate. In the event of imbalance between groups at baseline, regression analysis will be used to correct for the effect of the covariates.

The economic evaluation will address the question of whether or not immediate catheter drainage in patients with infected necrosis is cost-effective compared to the current management of postponed catheter drainage. A cost-effectiveness analysis and a cost-utility analysis will be performed, both from a societal perspective. The primary outcome parameters in the cost-effectiveness and cost-utility analyses, respectively, are the costs per unit of the CCI score and the costs per QALY.

### Monitoring and quality assurance

Clinical trial monitoring is performed by an independent monitor. The trial monitor checks and verifies documents and reports in the trial patients’ electronic or paper records at every site. The frequency may be changed based on the total enrollment period and enrollment rate. The monitor checks the site files according to the Medical Research Involving Human Subjects Act (WMO)/Good Clinical Practice (GCP) standards, as to whether and how essential documents are collected/administered and verifies all reported severe adverse events (SAEs). Also, the monitor verifies protocol compliance. A monitoring report is compiled after each monitoring visit at each specific site.

### Safety

Physicians who are involved in the trial will be asked to report all adverse events to the coordinating investigator. The independent Data Safety Monitoring Committee (DSMC) will evaluate safety parameters at regular intervals. The DSMC consists of five members: two surgeons, a gastroenterologist, a radiologist and an epidemiologist. Evaluations are planned after patient number 25, 50 and 75 have completed their 6-month follow up. Deceased patients and every SAE that is possibly trial-related will be discussed unblinded by the DSMC, 25 patients at a time in 4 sessions. During the inclusion period of the study the DSMC performs interim analyses only on safety. Only for safety reasons as assessed by the DSMC will the POINTER trial be prematurely terminated. There will be no interim analysis of treatment effect. Adverse events are reported using the online module (https://www.toetsingonline.nl) of the Dutch Central Committee on Research involving human subjects.

## Discussion

Infected necrosis is a potentially lethal complication of acute pancreatitis, typically requiring invasive intervention. The treatment of infected necrotizing pancreatitis is associated with lengthy hospital stay and high costs. The POINTER trial is the first randomized controlled trial designed to determine the optimal timing of catheter drainage in infected necrotizing pancreatitis: i.e. immediate or postponed, once walled-off necrosis has occurred.

According to current evidence-based international guidelines [7, 8], suspected or proven infected necrosis in patients with clinical signs of infection is an indication for invasive intervention. There should be a strong reluctance towards intervening in sterile collections [7, 28]. Currently, intervention is advised when the infected necrosis has become walled-off, which occurs typically 4 weeks after the onset of disease [29]. This practice of postponing interventions until the stage of walled-off necrosis is based on literature published when open necrosectomy was the standard intervention [10, 30, 31]. Early necrosectomy is now recognized as having a major impact in the critically ill patient, whereas postponed necrosectomy allows the immune system to recover from the pro-inflammatory response due to pancreatitis.

Since the step-up approach is now considered standard of care, the issue of the optimal timing of catheter drainage has become highly relevant. Current literature reports that 35–64% of patients with infected necrotizing pancreatitis can be treated with catheter drainage alone, without the need for invasive necrosectomy [3, 5]. Therefore, several expert pancreatologists have stated that they already practice immediate catheter drainage in patients with infected necrosis. In a recent international survey performed in preparation for this study, 55% of expert pancreatologists stated that they typically postpone catheter drainage by using antibiotics, whereas the other 45% proclaimed to drain immediately after diagnosing infected necrosis [17]. Thus, in practice immediate catheter drainage is already being performed in individual patients, regardless of the effect of antibiotics alone or the degree of encapsulation: (percutaneous) catheter drainage in infected necrosis has been described retrospectively in several cohorts [32] at a median of 2 weeks (9–15 days) after the onset of disease instead of at 4 weeks (28 days).

Patient outcomes were significantly better in the step-up-approach arm of the PANTER trial compared to the control group (primary open necrosectomy), but mortality (19%) did not differ between the two groups [3]. The POINTER trial assesses whether early detection of infected necrosis and immediate catheter drainage improve outcomes, to further reduce mortality and morbidity. Earlier intervention may prevent patients from further deterioration while often waiting several weeks before undergoing invasive intervention, and thereby reduce complications and length of hospital stay and improve patient quality of life. In the control group, the effect of antibiotics is awaited while letting the (extra) pancreatic necrotic collections become walled-off. Antibiotic treatment alone may suffice in a small minority of patients [4], which is another possible benefit for the control group.

Infected necrosis can be diagnosed on imaging (e.g. CECT) by the presence of gas in the (extra) pancreatic necrotic collection, irrespective of the source of the gas (i.e. through gas-forming bacteria or loss of integrity of the gastrointestinal tract). Collections with gas are seen in up to 42% of patients with infected necrosis [33] and can occur in every phase of the disease [34, 35]. Infected necrosis can also be confirmed by a positive gram stain or culture gathered with FNA. In a recent study, infected necrosis was confirmed by FNA in 86% (of 28 patients), which was similar to diagnosis based on clinical symptoms (80% of 92 patients) or gas identified on imaging (94% of 88 patients) [33]. Until recently, FNA was not routinely used in the Netherlands for diagnosing infected necrosis [17], as its outcome did not influence treatment because invasive intervention was postponed until the stage of walled-off necrosis, even in the case of a positive culture. In the POINTER trial, however, it is pivotal to detect infected necrosis as early as possible so as to perform immediate drainage (group A). Differentiating SIRS from sepsis is, however, very difficult in the first 14 days of the disease. Therefore, in the absence of gas on imaging but with clinical signs of infection in the first 14 days, a positive gram stain or culture after FNA is obligatory prior to randomization. Since the false negative rate of FNA is relatively high [33], a second FNA is advised in patients with persistent deterioration and a primary negative FNA. Clinical signs alone are sufficient to diagnose (suspected) infected necrotizing pancreatitis after the first 14 days [3].

After the first 14 days, clinical signs of infected necrosis are much more reliable. In the PANTER trial [3] it was possible to attain 91% accuracy in the identification of infected necrosis based on clinical criteria. Patients can be randomized in the POINTER trial after the first 14 days, based on the clinical diagnosis of infected necrosis, as was done in the PANTER trial [3].

Patients are randomized to undergo either immediate (< 24 h) or postponed (in walled-off necrosis) catheter drainage of infected collections. Both the surgical and the endoscopic step-up approach are allowed, depending on the location of the necrotic collection(s), the extent of encapsulation and the preference of the treating physician. It is known that both approaches are effective and safe and that not all (peri) pancreatic collections are approachable using a single technique [6, 9].

The CCI [20, 21] is the primary endpoint of the trial. Patients with infected necrotizing pancreatitis often have a long disease course with multiple complications, and therefore the CCI score is considered a representative tool to take into account all these complications. The individual complications (e.g. organ failure and mortality), number of (repeat) interventions, hospital and ICU lengths of stay, QALYs and direct and indirect costs are also analyzed as secondary end points.

Patients are stratified based on organ failure at baseline, since it is known that patients with organ failure have poorer outcomes, as compared to patients with no organ failure. Patients are also stratified on disease duration (cutoff 20 days) since this is obviously related to the onset of walled-off necrosis. Finally, patients are stratified on expected high versus low volumes of patient inclusion.

In conclusion, the POINTER trial is a multicenter randomized controlled trial that investigates whether immediate catheter drainage reduces the CCI in patients with infected necrotizing pancreatitis, as compared to postponed catheter drainage.

## Trial status

The trial was registered on 6 August 2015 as ISRCTN33682933 (http://www.isrctn.com/ISRCTN33682933). The first patient was randomized on 4 August 2015. To date, 88 of the 104 patients have been randomized and the inclusion of patients is on schedule.

## Additional files

Additional file 1: SPIRIT checklist. (DOCX 46 kb)

Additional file 2: **Table S1.** Inclusion and exclusion criteria. (DOCX 14 kb)

Additional file 3: **Table S2.** Criteria for infected necrotizing pancreatitis. (DOCX 14 kb)

Additional file 4: **Figure S1.** Inclusion and randomization flowchart. (DOCX 33 kb)

Additional file 5: **Figure S2.** Surgical step-up approach [32, 37, 38]. (DOCX 253 kb)

Additional file 6: **Figure S3.** Endoscopic step-up approach [32, 37, 38]. (DOCX 169 kb)

Additional file 7: Relevant definitions. (DOCX 17 kb)

## Abbreviations

APA: American Pancreatic Association
APACHE: Acute Physiology and Chronic Health Evaluation
ASA: American Society of Anesthesiologist’s classification
CCI: Comprehensive Complication Index
CECT: Contrast enhanced computed tomography
CRP: C-reactive protein
CTSI: Computer Tomography Severity Index
DPSG: Dutch Pancreatitis Study Group
DSMC: Data Safety Monitoring Committee
FNA: Fine needle aspiration
GCP: Good Clinical Practice
IAP: International Association of Pancreatology
ICU: Intensive care unit
iMCQ: iMedical Consumption Questionnaire
iPCQ: iProductivity Cost Questionnaire
MODS: Multiple Organ Dysfunction Score
MRI: Magnetic resonance imaging
QALY: Quality-adjusted life year
SAE: Serious adverse event
SD: Standard deviation
SIRS: Systemic inflammatory response syndrome
SOFA: Sequential Organ Failure Assessment
SSI: Surgical site infection
VARD: Videoscopic assisted retroperitoneal debridement
WMO: Medical research involving human subjects act (in Dutch, *Wet Medisch-wetenschappelijk Onderzoek met mensen*)
