# Supplementary material for: Postponed or immediate drainage of infected necrotizing pancreatitis (POINTER trial): study protocol for a randomized controlled trial
Source: Trials. 2019 Apr 25;20:239. doi: 10.1186/s13063-019-3315-6 (PMC6482524; doi:10.1186/s13063-019-3315-6)
Supplement: Supplementary file 2 — Table S1. Inclusion and exclusion criteria. (DOCX 14 kb) [file 13063_2019_3315_MOESM2_ESM.docx]

Additional file 2: Table S1: in- and exclusion criteria

| **Inclusion** | **Exclusion** |
| --- | --- |
| Infected necrotizing pancreatitis^1^  Day 0-35: gas on imaging or positive culture  Day 15-35: clinical signs alone allowed | Onset of acute pancreatitis >35 days ago |
| Catheter drainage of the necrotic collection is technically feasible^2^ | Indication for emergency laparotomy because of abdominal catastrophe^3^ |
| Age ≥ 18 years | Previous retroperitoneal intervention for necrotizing pancreatitis^4, 5^ |
|  | Documented chronic pancreatitis^6^ |
|  | |
| *^1^ See criteria in table S2*  *^2^ As deemed by the expert panel and/or treating physician (i.e. enough encapsulation and liquefaction)*  *^3^ For example bleeding, bowel perforation or abdominal compartment syndrome*  *^4^ Ascites drainage is permitted*  *^5^ Emergency laparotomy without opening the bursa is permitted*  *^6^ According to the M-ANNHEIM criteria [36]* | |
