# Supplementary material for: Postponed or immediate drainage of infected necrotizing pancreatitis (POINTER trial): study protocol for a randomized controlled trial
Source: Trials. 2019 Apr 25;20:239. doi: 10.1186/s13063-019-3315-6 (PMC6482524; doi:10.1186/s13063-019-3315-6)
Supplement: Supplementary file 3 — Table S2. Criteria for infected necrotizing pancreatitis. (DOCX 14 kb) [file 13063_2019_3315_MOESM3_ESM.docx]

Additional file 3: Table S2: criteria for infected necrotizing pancreatitis

| **Day 0-14 after onset of disease:**  **proven infected necrosis** | **Day 15-35 after onset of disease:**  **proven or suspected infected necrosis** |
| --- | --- |
| Gas in the necrotic collection on imaging | Gas in the necrotic collection on imaging |
| Positive gram stain/culture of fine-needle aspiration from the necrotic collection | Positive gram stain/culture of fine-needle aspiration from the necrotic collection |
|  | Clinical signs of infection without obvious another focus than infected necrosis for 3 consecutive days^7^ |
|  | |
| *^7^ Either persistent (multiple) organ failure in patients admitted to the Intensive Care Unit. Or 2 of the 3 inflammatory parameters not decreased (temperature (T>38.5 ⁰C), C-Reactive Protein or leukocyte count) during 3 consecutive days in patients on regular wards (with no other infection focus). These clinical criteria alone are considered sufficiently reliable only after the initial 14 days of acute pancreatitis.* | |
