# Supplementary material for: Postponed or immediate drainage of infected necrotizing pancreatitis (POINTER trial): study protocol for a randomized controlled trial
Source: Trials. 2019 Apr 25;20:239. doi: 10.1186/s13063-019-3315-6 (PMC6482524; doi:10.1186/s13063-019-3315-6)
Supplement: Supplementary file 4 — Figure S1. Inclusion and randomization flowchart. (DOCX 33 kb) [file 13063_2019_3315_MOESM4_ESM.docx]

Additional file 4: Figure S1: inclusion and randomization flowchart

If needed, minimally invasive necrosectomy

If needed, drain revising/upsizing

If needed, catheter drainage (preferably in walled-off necrosis)

Antibiotics

**B**

If needed, minimally invasive necrosectomy

If needed, drain revising/upsizing

Antibiotics and <24h catheter drainage

**A**

**RANDOMIZATION POINTER**

Catheter drainage is technically feasible

Suspected infected necrosis

Proven infected necrosis

Gas in necrotic collection

Positive culture or gram stain after fine needle aspiration

Clinical signs of infection without another focus than infected necrosis

Day 0-14 after onset of disease

Day 15-35 after onset of disease

**Patient with necrotizing pancreatitis**
