# Supplementary material for: Postponed or immediate drainage of infected necrotizing pancreatitis (POINTER trial): study protocol for a randomized controlled trial
Source: Trials. 2019 Apr 25;20:239. doi: 10.1186/s13063-019-3315-6 (PMC6482524; doi:10.1186/s13063-019-3315-6)

Additional file 5: Figure S2: surgical step-up approach [32]

A| Cross-sectional image and torso depicting a peripancreatic collection with fluid and necrosis. The first step of the surgical step‑up approach is percutaneous catheter drainage. The preferred access route is through the left retroperitoneal space between the left kidney, dorsal spleen and descending colon. If necessary, percutaneous catheter drainage is followed by a minimally invasive surgical necrosectomy, for example videoscopic-assisted retroperitoneal debridement. B| Enlargement of the area of detail shown in part a of the figure. C| A 5 cm subcostal incision is made, and the previously placed percutaneous drain is used as a guide into the retroperitoneum to enter the necrotic collection. The first necrosis is removed under direct vision with a long grasping forceps. D| Further debridement is performed under videoscopic assistance.

*Reprinted from van Brunschot, S. et al. Clin. Gastroenterol. Hepatol. 10, 1190-1201 (2012) [37], with permission from Elsevier ©, and adapted from John Wiley and Sons © da Costa, D. W. et al. Br. J. Surg. 101, e65-e79 (2014) [38].*


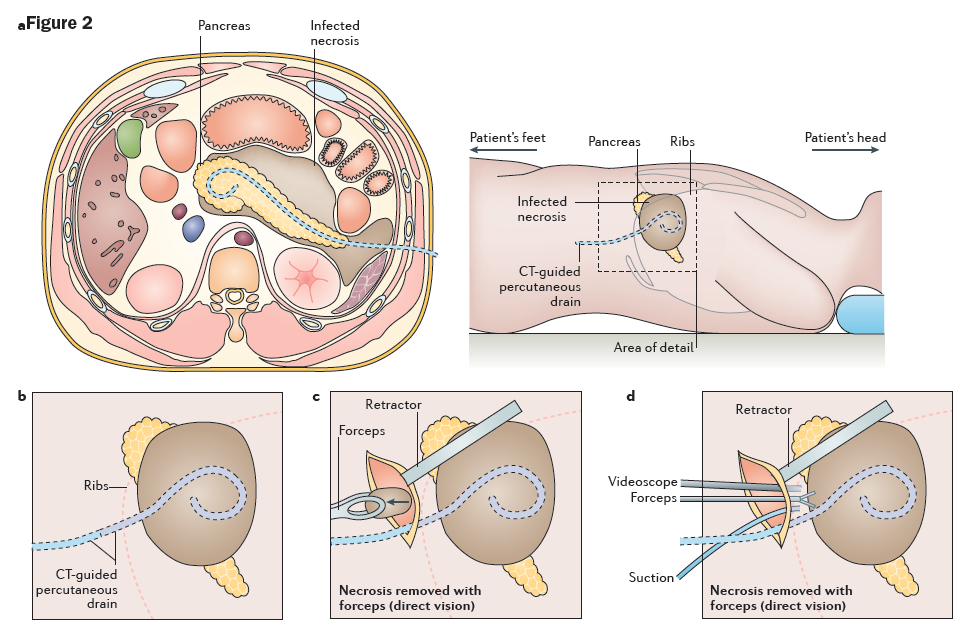

Supplement: Supplementary file 5 — Figure S2. Surgical step-up approach [32, 37, 38]. (DOCX 253 kb) [file 13063_2019_3315_MOESM5_ESM.docx]
