# Supplementary material for: Postponed or immediate drainage of infected necrotizing pancreatitis (POINTER trial): study protocol for a randomized controlled trial
Source: Trials. 2019 Apr 25;20:239. doi: 10.1186/s13063-019-3315-6 (PMC6482524; doi:10.1186/s13063-019-3315-6)

Additional file 6: Figure S3: endoscopic step-up approach [32]

A| The first step of the endoscopic step‑up approach is endoscopic transluminal drainage. The preferred access route for endoscopic transluminal treatment is through the posterior wall of the stomach. The necrotic collection often bulges into the stomach, facilitating endoscopic transluminal treatment. The collection is punctured through the gastric wall, followed by balloon dilatation of the tract. Two double-pigtail stents and a nasocystic catheter are placed for continuous postoperative irrigation. B| If necessary, the cystostomy tract is further dilated, the collection is entered by a forward viewing endoscope, and necrosectomy is performed.

*Reprinted from van Brunschot, S. et al. Clin. Gastroenterol. Hepatol. 10, 1190-1201 (2012) [37], with permission from Elsevier ©, and permission obtained from John Wiley and Sons © da Costa, D. W. et al. Br. J. Surg. 101, e65-e79 (2014 [38]).*


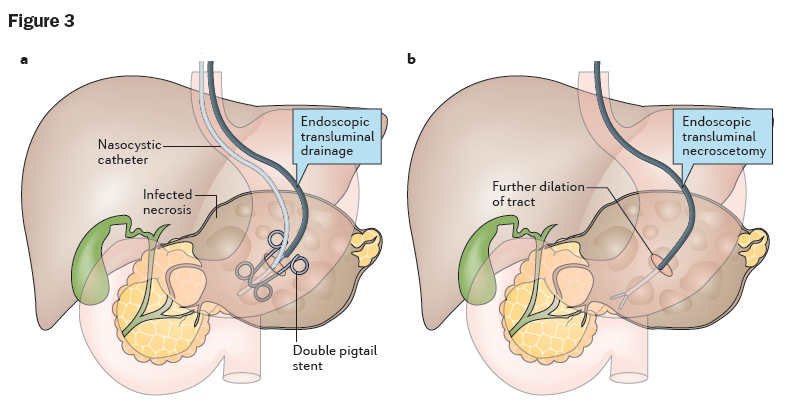

Supplement: Supplementary file 6 — Figure S3. Endoscopic step-up approach [32, 37, 38]. (DOCX 169 kb) [file 13063_2019_3315_MOESM6_ESM.docx]
