# Supplementary material for: Postponed or immediate drainage of infected necrotizing pancreatitis (POINTER trial): study protocol for a randomized controlled trial
Source: Trials. 2019 Apr 25;20:239. doi: 10.1186/s13063-019-3315-6 (PMC6482524; doi:10.1186/s13063-019-3315-6)
Supplement: Supplementary file 7 — Relevant definitions. (DOCX 17 kb) [file 13063_2019_3315_MOESM7_ESM.docx]

Additional file 7: Relevant definitions

**Acute pancreatitis**

Upper abdominal pain and serum amylase and/ or lipase >3x upper limit of normal,

potentially with cross-sectional imaging if first two criteria unclear.

**Endocrine pancreatic insufficiency**

The need for insulin or oral anti-diabetic drugs; this requirement was not present before onset of pancreatitis.

**Enterocutaneous fistula**

Secretion of fecal material from a percutaneous drain or drainage canal after drain removal

or from a surgical wound, either from small or large bowel; confirmed by imaging or during

surgery.

**Exocrine pancreatic insufficiency**

Fecal elastase <0.2 gram and the need for pancreatic enzyme supplementation; this requirement was not present before onset of pancreatitis.

**Incisional hernia**

Incisional hernia is defined as full-thickness discontinuity in abdominal wall and bulging of abdominal contents, with or without obstruction.

**Intra-abdominal bleeding**

Bleeding requiring surgical, radiologic, or endoscopic intervention.

**M-ANNHEIM criteria**

One or more of the following criteria [36]:

1) Pancreatic calcifications

2) Moderate or marked ductal lesions

3) Marked and persistent exocrine insufficiency defined as pancreatic steatorrhea markedly reduced by enzyme supplementation

4) Typical histology of an adequate histological specimen

**Multiple Organ Dysfunction Score (MODS)**

Scale that ranges from 0 to 24, with higher scores indicating more severe organ dysfunction [26].

**Multiple organ failure**

Failure of 2 or more organ systems (respiratory, cardiovascular or renal) occurring in the same 24 hours.

**Necrotizing pancreatitis**

Either pancreatic necrosis or extrapancreatic necrosis. Pancreatic necrosis is defined as diffuse or focal area(s) of non-enhancing pancreatic parenchyma as detected on CECT. Extrapancreatic necrosis is defined as persistent peripancreatic fluid collections on CECT in the absence of pancreatic parenchymal non-enhancement.

**New onset (multiple) organ failure**

Failure of one (or more) organ systems that was not present prior to randomization or at any time in the 24 hours before intervention.

**Organ failure**

Failure of one or more of the following organ systems (adapted from the Atlanta classification) and as previously used in the PANTER and TENSION trial [3, 9]:

1) Respiratory: PaO2 <60 mm Hg despite FiO2 30% or the need for mechanical ventilation (pulmonary insufficiency)

2) Cardiovascular: systolic blood pressure <90 mm Hg despite adequate fluid resuscitation or the need for vasopressor support (cardiocirculatory insufficiency)

3) Renal: serum creatinine level >177 umol/L after rehydration or the need for hemofiltration or hemodialysis (renal failure) (in case patients already suffered from renal insufficiency before this episode of AP [creatinine >177 umol/L] this does not count as renal failure)

**Pancreaticocutaneous fistula**

Output through a percutaneous drain or drainage canal after removal of drains from a surgical wound, or any measurable volume of fluid with an amylase content >3 times the serum amylase level.

**Persistent organ failure**

Failure of one or more organ systems for at least 48 hours.

**Proven infected necrosis**

A positive culture or gram stain obtained by fine-needle aspiration from the necrotic collection or gas configurations in the necrotic collection on imaging.

**Perforation of visceral organ**

Perforation requiring surgical, radiologic, or endoscopic intervention.

**Sequential Organ Failure Assessment (SOFA) score**

Scale that ranges from 0 to 24, with higher scores indicating more severe organ dysfunction [27].

**Suspected infected necrosis**

Either persistent (multiple) organ failure in patients admitted to the Intensive Care Unit. Or 2 of the 3 inflammatory parameters not decreased (temperature (>38.5 ⁰C), C-Reactive Protein or leukocyte count) during 3 consecutive days in patients on regular wards (with no other infection focus). These clinical criteria alone are considered sufficiently reliable only after the initial 14 days of acute pancreatitis.

**Wound infection**

Defined as a superficial incisional surgical site infection (SSI) and must meet the following criterion: infection occurs within 30 days after the operative procedure and involves only skin and subcutaneous tissue of the incision and the patient has at least 1 of the following:

1) Purulent drainage from the superficial/deep incision but not from the organ/space component of the surgical site

2) Organisms isolated from an aseptically obtained culture of fluid or tissue from the superficial incision

3) At least 1 of the following signs or symptoms of infection: pain or tenderness, localized swelling, redness, or heat, and superficial incision is deliberately opened by surgeon and is culture positive or not cultured. A culture-negative finding does not meet this criterion.

4) An abscess or other evidence of infection involving the deep incision is found on direct examination, during reoperation, or by histopathologic or radiologic examination

5) Diagnosis of superficial/deep incisional SSI by the surgeon or attending physician
